# Aerodynamic Performance of a Dragonfly-Inspired Tandem Wing System for a Biomimetic Micro Air Vehicle

**DOI:** 10.3389/fbioe.2022.787220

**Published:** 2022-05-18

**Authors:** Erfan Salami, Elham Montazer, Thomas A Ward, Nik Nazri Nik Ghazali, Irfan Anjum Badruddin

**Affiliations:** ^1^ Department of Mechanical Engineering, Faculty of Engineering, University of Malaya, Kuala Lumpur, Malaysia; ^2^ Department of Mechanical Engineering, Sharif University of Technology, Tehran, Iran; ^3^ School of Engineering and Computer Science, Cedarville University, Cedarville, OH, United States; ^4^ Research Center for Advanced Materials Science (RCAMS), King Khalid University, Abha, Saudi Arabia; ^5^ Department of Mechanical Engineering, College of Engineering, King Khalid University, Abha, Saudi Arabia

**Keywords:** bioinspired, dragonfly, unsteady aerodynamics, biomimetic micro air vehicle, tandem flapping wings

## Abstract

The flying agility demonstrated by dragonflies is accomplished by means of complex aerodynamic forces produced by flapping their four wings arranged in a tandem configuration. The current study presents a novel tandem flapping wing mechanism for a biomimetic air vehicle that was designed and manufactured to experimentally investigate the aerodynamic forces. By optimizing the configuration and using spatial network analysis, it is shown that the designed structure can flap the wings in a linear up–down stroke motion and is capable of maintaining good consistency and aerodynamic performance. Such a mechanism could be used in a future biomimetic micro air vehicle (BMAV) design. The mechanism uses an electromagnetic actuator to flap the wings with a variable beat frequency (30–210 Hz) at various angles of attack (−10°–20°). The results show that the tandem wings generate approximately 50% higher lift than the forewing or hindwing pairs acting alone. Tandem wings also improve stability, which could potentially allow hovering.

## Introduction

Biomimetic micro air vehicles (BMAVs) are a group of microsized, unmanned aircraft that are bioinspired by the flapping wing motion of flying small birds or insects. Their microscaled size and ultra-light weight nature allow them to potentially fly inside buildings or compact spaces, making them the ideal urban drone. They can be used for remote surveillance of hazardous sites such as chemical spills, radiation leaks, high-voltage areas, active crime scenes, or other dangerous areas.

In order to understand the interest in BMAVs, it helps in having a better understanding of their aerodynamics (Salami et al., 2019). Insects use many unsteady and complex wing motions to generate lift. Ansari et al. were able to create models for the figure-of-eight motion that accounts for both the steady-state lift and unsteady lift component. The latter was broken down into leading-edge vortex (LEV) and trailing-edge wake (Ansari et al., 2006a; Ansari et al., 2006b). There are many types of lifts that insects use that can be mimicked by BMAVs (Li et al., 2021).

Dragonflies are quick flyers (Salami et al., 2016; Salami et al., 2017; Sivasankaran et al., 2017) that can change course quickly and move in a different direction. They can rapidly speed up from a hover and vice versa. They have a high power-to-weight ratio because of the low aspect ratio of their wings. Rotating wings about several axes is another ability of dragonflies. This makes them among the quickest and most agile flying insects in the world (Li et al., 2018). The complex structure of dragonfly wings plays the main role in this capability (Rubentheren et al., 2016; Zhang et al., 2018; Salami et al., 2020).

Numerous scientists have studied the aerodynamics produced by various wing beat frequencies, wing motions, or interactions between the wings of dragonflies since they are very common and distributed almost around the world. Several studies have looked at the effects of subtle wing structural modifications on aerodynamics. The aforementioned topics are crucial in grasping when applying these features to future BMAV developments.

Flight with microscaled wing surface areas is possible because of the additional lift gained by flapping the wings. Lift and thrust are achieved due to vorticities generated by this flapping wing motion. One of the first types of flapping wing motion studied is clap-and-fling kinematics. These kinematics studies are typical of butterflies and hummingbirds (Weis-Fogh, 1972). Lift is generated as a wing pair flap and comes together at the end of a half stroke (clap) and then flings apart (similar to quickly flinging a hard-bound book open and closed) (Weis-Fogh, 1973). This motion generates a bound vortex on each wing that plays as the initial vortex of the opposite wing.

While the clap-and-fling was the first unsteady aerodynamic lift motion to be well-understood, further work by Lighthill, Maxworthy, and Ellington built upon this early study of insect flight (Lighthill, 1973; Maxworthy, 1979; Ellington, 1984). Ellington et al. discovered a wing motion known as the LEV (Ellington et al., 1996). Lift is generated by vortices attached to the wing as the wing pitches to an angle of attack (AOA) beyond the stall angle (for a conventional airfoil). This was further verified and quantified by several aerodynamicists by computational fluid dynamics (CFD) studies (Liu et al., 1998; Lan and Sun, 2001; Sun and Tang, 2002). Dickinson called the LEV generated by a translating wing “delayed stall” (Truong et al., 2011). Dickenson used a reduced order model to determine the role of the LEV and other wing kinematics in the total lift. By placing a large aphid wing model with six computer-controlled stepper motors used to flap the wing in mineral oil, it could be determined how the lift was generated by different wing motions (Dickinson et al., 1999). It was concluded that the primary contributor to the lift during hover is LEV-generated, but it was also found that more than 35% of the total lift generated was due to rotational effects (Dickinson et al., 1999).

Another significant component of the lift noted was wake capture. Wake capture is the lift generated when the wing changes direction and utilizes the wake from the previous flapping half stroke to generate lift. This is similar to the lift generated when an aircraft takes off into the wind, utilizing the flow of the wind over the wing to generate additional lift. Sun and Tang verified the additional lift generated at the end of each half stroke and the beginning of the next half stroke using CFD but offered different explanations for their occurrence (Sun and Tang, 2002).

Walker explained that since the Magnus force is independent of AOA and the rotational moment, it could not be the force Dickinson described (Walker, 2002). Walker also made a strong case for the inclusion of wake capture. After this, Dickinson stopped describing this as a Magnus force–like effect, revising his terminology to name it “rotational circulation” (Sane and Dickinson, 2002). More current research studies have identified the following sources of insect unsteady aerodynamic lift: clap-and-fling, LEV or delayed stall, added mass inertia (rotational inertia), rotational circulation, and wake capture (Sun and Xiong, 2005; Truong et al., 2011). Of these, added mass inertia is small and usually included as part of the rotational circulation and clap-and-fling is used by only a few flying insects or birds (Karásek, 2014). This leaves LEV, rotational circulation, and wake capture as the primary types of unsteady aerodynamic lift.

Early benchtop mechanisms focused on generating appropriate flight kinematics by flapping the wings in a figure-of-eight flapping translational motion while also variably rotating (or pitching) the wings (Conn et al., 2007; Galiński and Żbikowski, 2007). Many mechanisms were used to validate aerodynamic models of lift generation (Dickson and Dickinson, 2004; Galiński and Żbikowski, 2005). Based upon the hover model, turning using aerodynamic damping has also been studied (Cheng and Deng, 2011). Still, others have focused on generating flapping flight, but most of these BMAVs use a sail-like membrane to generate lift, which undulates rather than truly flaps (De Croon et al., 2009; Tsai and Fu, 2009; Hsu et al., 2010).

The wing structure is also very important. Wings must be able to manage the forces generated by flapping while remaining ultra-lightweight (Sivasankaran et al., 2016). The wings must be fabricated from advanced lightweight materials. Wings using chitin nanocomposite films, bioinspired from insect wing membranes, can be used to reduce the overall weight (Rubentheren et al., 2016).

Bomphrey and Nakata (Bomphrey et al., 2016) studied the aerodynamic performance of real dragonfly wings and its comparison with artificial wings by providing detailed 3D wing geometries of dragonflies and performing CFD and PIV analysis. They performed simulations at various speeds and angles of attack. They reported that the forewing sits in a region of positive pressure generated by the hindwing and, therefore, experiences reduced drag; conversely, the hindwing suffers higher drag owing to the forewing.

Accordingly, the aerodynamics comparison study among the selected BMAV wing configuration is still lacking. Therefore, the overall purpose of the present investigation is to compare the aerodynamic performances (lift, drag, and lift-to-drag ratio) between the symmetrical BMAV forewing, hindwing, and tandem wings at various wing beat frequencies and angles of attacks. Based on the experimental force measurements and high-speed imaging, the impact of wing configuration on the aerodynamic performance of the BMAV flapping flight is explained in the *Discussion.* In the time domain where cyclic averaging is being used to enhance the force profile, the stronger force peaks occur when the wing is generating lift at specific points during the flapping. Finally, the aerodynamic performance results obtained in this study are validated by real dragonfly data benchmarks taken from Bomphrey and Nakata (Bomphrey et al., 2016). Since future operational BMAV will likely be disposable due to their utility in conducting one-way missions into hazardous environments, minimizing cost and fabrication time is important. Therefore, utilizing lightweight disposable 3D printed plastic construction is a secondary goal of this research.

## Aerodynamic Model

### BMAV Wing

Since it is very complicated and expensive to manufacture the complex microstructures of a real dragonfly wing, the created wings are simplified duplications of the real wing set. The spatial network analysis technique has been used to perform the simplification. This method has performed the segmentation based on the pattern density value in order to bio-mimic the real dragonfly wing (Sivasankaran et al., 2016). An initial wire frame, 2D scaled model, was manufactured utilizing SolidWorks to build a 3D solid model by extruding the imported 2D wire frame.

The experimental results of the authors’ previous research (Salami et al., 2020) regarding the nanomechanical properties of various BMAV wing materials demonstrated that the HIPS (high-impact polystyrene) and Ultrat fabricated wings approximately resembled a real dragonfly’s nanoindentation results. While fabricating the BMAV with a 3D printer, it was found that HIPS had better final finishing and it is easier to take a more consistent melting temperature using the MakerBot machine. Accordingly, HIPS was chosen to fabricate the BMAV wings for the present study.

**FIGURE 1:**
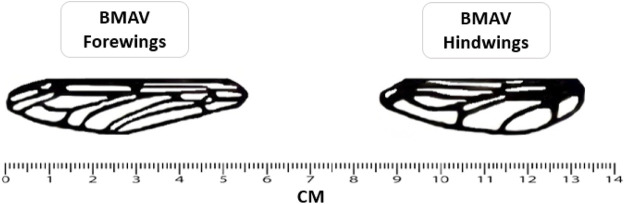
Fabricated wings utilized in the present study.

A synthesized, thin-film, nanocomposite membrane was then laid down over the wing frames. Figure 1 and Table 1 show the final BMAV fabricated wings and wing structure specifications.

**TABLE 1:** BMAV wing structure specification.

Specification	Forewing	Hindwing
Base width	7.00 mm	8.5 mm
Center width	12.50 mm	14.4 mm
Tip width	7.23 mm	8.5 mm
Length	56.00 mm	48.00 mm
Mass	0.12 g	0.08 g

**FIGURE 2:**
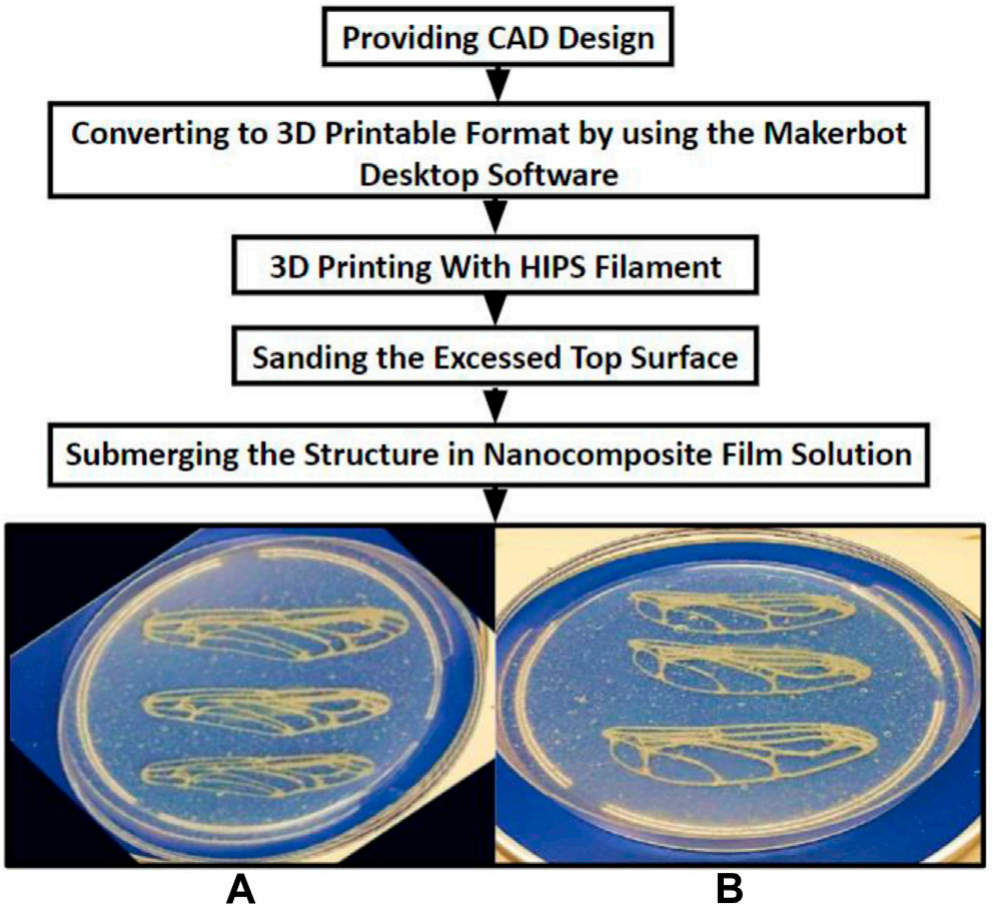
Design process: **(A)** Forewing, **(B)** Hindwing.

Once the wing structure was printed out, the exposed top surface layer required sanding to remove the jagged material remains on the structure. The structures were then submerged in the selected chitosan nanocomposite film solution in a petri dish, as illustrated in Figure 2. The chitosan nanocomposite suspension constituted a chitosan suspension reinforced with nanosized whiskers and crosslinked using tannic acid. The suspension was transformed into a thin 3-mm film using the casting evaporation method. A drying time of 48 h was required.

### Flapping Mechanism and Experimental Setup

**FIGURE 3:**
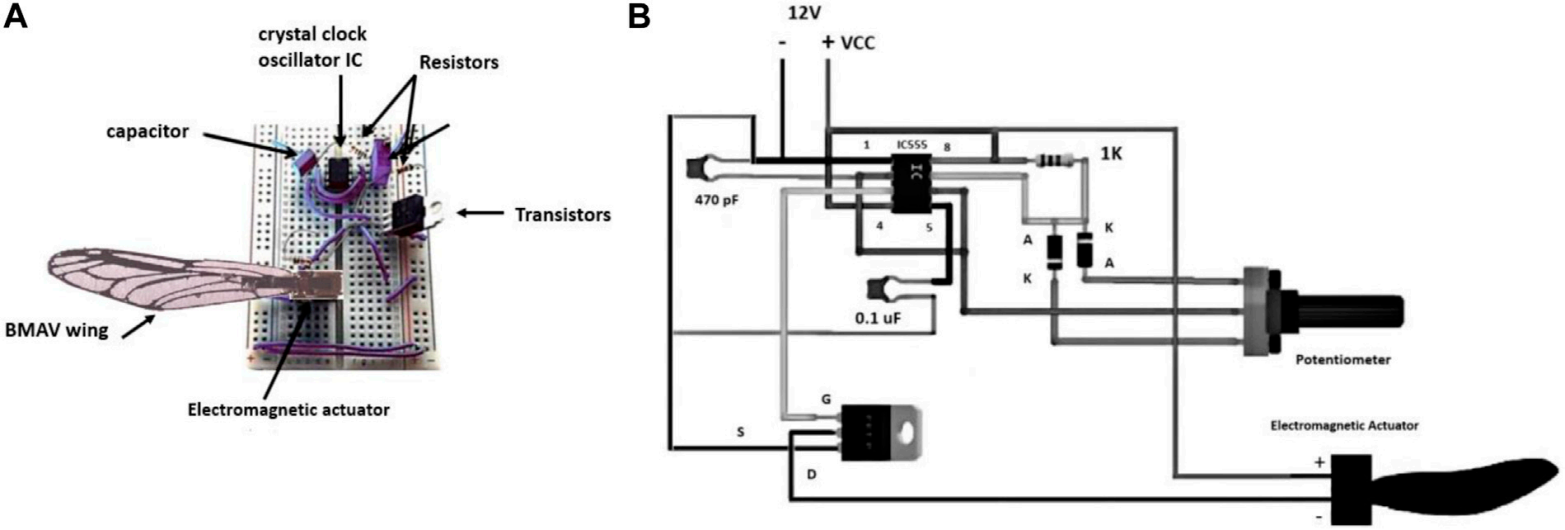
Flapping mechanism: **(A)** Actual setup, **(B)** Electronic overview diagram.

An electromagnetic flapping wing actuator has been utilized as a wing flapping mechanism in the current study. The power supply applied in this flapping wing mechanism was 12 V DC. For having a stable oscillation, an LM555 crystal clock oscillator integrated circuit, which has been displayed in Figure 3, was utilized. A capacitor and two resistors were used to accurately control the free-running frequency and duty cycle. The generated oscillation was fed to a Power MOSFET fast switch. The output of the Power MOSFET was utilized to actuate the miniature PC Board Relay. The frequency of the switch (corresponding to the wing beat frequency) can be adjusted by a 22-k potentiometer. A superglue was used to attach each of the BMAV wings to a flat iron plate (2 mm long and 2.75 mm thick) using. The iron plate was oscillated by an electromagnetic actuator (3–3 mm).

Figure 3 displays the flapping mechanism setup and electrical diagram of a wing structure attached to the actuator. For mimicking the joint of the real dragonfly, the plate was attached to the hinge of the BMAV wing. This flapping mechanism can make a linear up–down stroke motion at different wing beat frequencies, up to a maximum frequency of 250 Hz. Based on the real dragonfly wing flapping angle during hovering flight, the flapping degree was fixed to be 90°.

## BMAV Flapping Aerodynamics

Testing of the electromagnetic flapping mechanism was broken down into experiments and time-frequency domain analysis of the experimental data using cyclic averaging and fast Fourier transform (FFT). The frequency was varied by adjusting a power output voltage over the test run. Force data samples were collected at each frequency. Force data points were averaged to a single point. The only way to adjust the wing beat frequency was the DC voltage applied to the actuators. The wing beat frequency was then changed by adjusting the voltage applied by the power supply.

**FIGURE 4:**
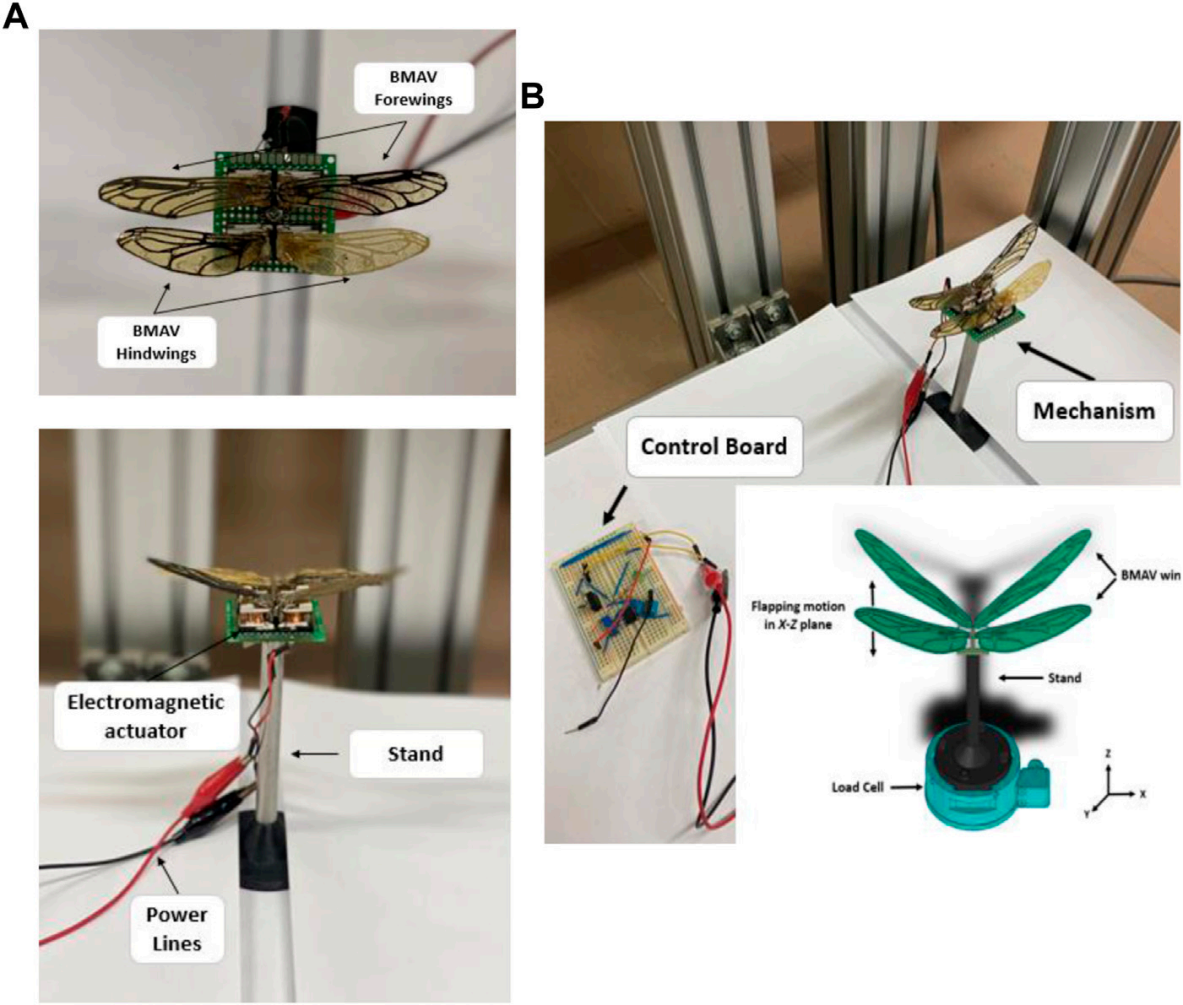
**(A)** Wings and actuator formation, **(B)** Experimental setup.

As Figure 4 displays, to measure the forces, the electromagnetic mechanism was placed on a test stand attached to an ATI Gamma six-axis force/torque sensor. The FFT low-pass filter was used to filter the raw data which were obtained by the sensor. The net aerodynamic force was finally achieved by deducting the gravitational and inertial forces obtained in air from the total forces measured.

The *z*-axis force data (lift) and the *y*-axis force data (drag) are of primary importance and are looked upon for further analysis and understanding of the flapping mechanism. Torque data were deemed to add no additional information than that available from the force data. The torque data were useful early on in making sure that the sensor was aligned properly. Short duration data collection runs were used because of the large amount of data being collected. Each force data point plotted was the average of 10,000 samples over 10 s. Each test run was conducted over multiple frequencies to make a plot. Each experiment included five test runs.

**FIGURE 5:**
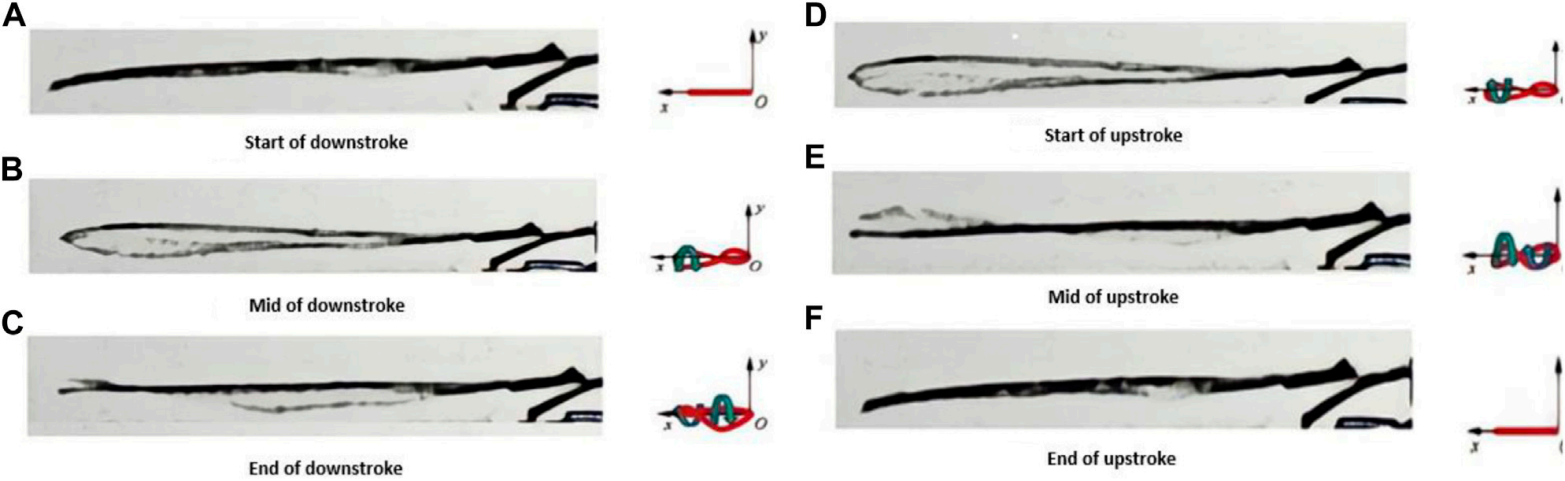
Side view of the flapping wing captured by high speed camera during one flapping cycle at 30 Hz **(A)** Start of downstroke, **(B)** Mid of downstroke, **(C)** End of downstroke, **(D)** Start of upstroke, **(E)** Mid of upstroke, **(F)** End of upstroke.

The frequency is consistently monitored directly from the control board during the experiment as the digital output is directly proportional to the supplied voltage. The secondary method of calculating the flapping frequency from the high-speed camera data through its slow-motion capability and using a timer gives another way to verify the flapping frequency, as shown in Figure 5. The voltage supplied to the control board is directly proportional to the flapping frequency, and if the voltage is constant, the flapping frequency is also constant. So, if the digital readout stays constant, the flapping frequency is held constant. This also means that the more power (related to the voltage by P = V^2^/R) delivered to the motor, the faster it flaps. The frequency can be increased proportionately by increasing the digital readout from one data collection to the next.

From a frequency-domain perspective, there will be peaks in the spectrum at each flapping wing cycle. The more consistent the flapping rate, the stronger the peak at that flapping rate or frequency. In the time domain where cyclic averaging is used to enhance the force profile, the stronger force peaks occur when the wing generates lift at specific points during flapping.

Since the data repeat at regular flapping intervals, the cyclical points of interest become more pronounced through cyclic averaging over multiple flapping periods, while more random data should be less pronounced. This means the vibrational noise data are random and can be reduced through averaging.

**FIGURE 6:**
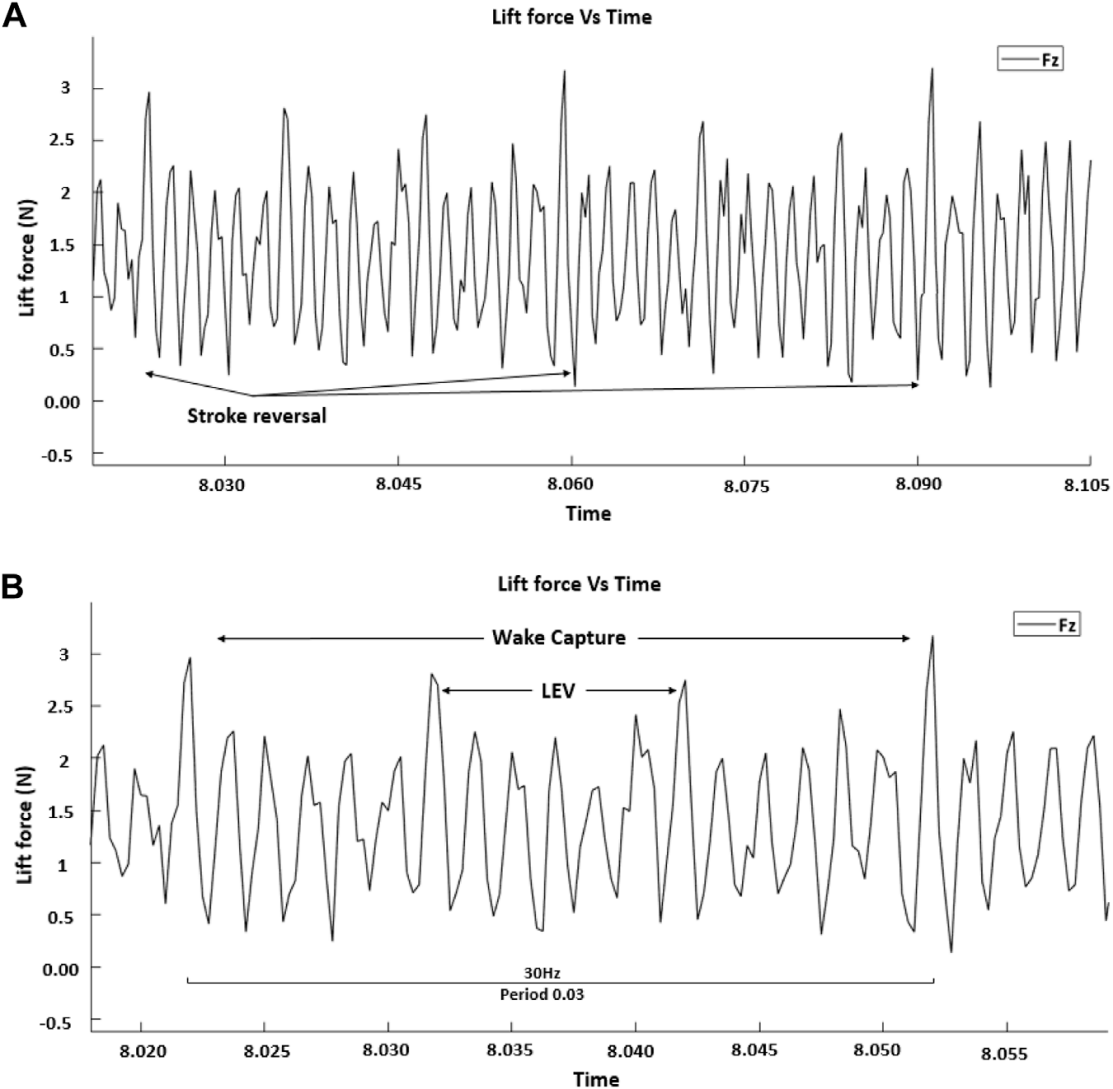
Cyclic averaged data showing **(A)** multi periods and **(B)** a single period.

According to Dickinson, a baseline for the LEV effects could be generated. It became apparent that there were significant effects at the beginning and end of the stroke for which there explanations were needed. The peak at the beginning of the flapping wing half stroke is considered to be due to wake capture. The LEV is the greatest contributor to lift and wake capture, producing significant portions of the additional lift. Figure 6 illustrates the averaged lift data for different periods of flapping cycles. There are also cyclical anomalies (vibrations) related to the flapping frequency that will become more pronounced and visible, especially as the frequency increases. Vibration anomalies can be seen in Figure 6, the smaller peaks in between LEV and wake capture peaks that are pointed by arrows. Reviewing the high-speed camera data shows that these readings are due to the wing going through mechanical oscillations.

The valley in between the two peaks that is pointed to by the arrows is the wing reversal for each half stroke, and the data between wake capture peaks in Figure 6 have a positive average and correspond to the lift from the LEV. The first peak in each wing flapping cycle is due to stroke reversal, and the peak at the end of each cycle is due to wake capture. The data represent two forewings flapping symmetrically at a frequency of 30 Hz, which relates to a period of 0.03 s. The lift profiles show that the lift generated has components of both wake capture and LEV.

## Aerodynamics Performance

The experimental parameters are set on the basis of a comparison of the results obtained from previous researchers and the limitations of the experimental setup. The BMAV flight has been influenced by AOA, wing beat frequency, Reynolds number, and freestream velocity.

The aerodynamic forces and aerodynamics performance were evaluated for the flapping wing configuration from −10° to 20° AOA at various wing beat frequency ranges from 30 to 210 Hz. The chord Reynolds case relating to Rec = 0 was chosen to be the static case which means no incoming freestream velocity. Each test was repeated five times, and the results are the average amounts of these five trials. The best way to adjust the wing beat frequency was the DC voltage applied to the actuators. The wing beat frequency was then varied by changing the voltage applied by the power supply. The voltage was increased by increments of 1.0 V from 5 to 12 V. Since the relationship between the wing beat frequency and corresponding applied voltage from the power supply is linear, the frequency changes slightly.

**FIGURE 7:**
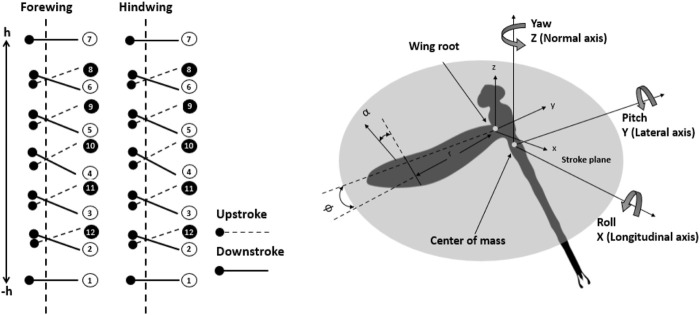
Time history position and angles of attack of both forewing and hindwing trajectories at zero phase differences.

Figure 7 shows the time histories of the positions and angles of attack of both wings. The rotational axis was at the quarter chord from the leading edge of the wing model. The distance between the two wings was fixed at 1.5 chord length of the forewing, which has been demonstrated to be fitting (Maybury and Lehmann, 2004; Bomphrey et al., 2016). The dragonflies had been recorded by Wakeling and Ellington (Wakeling and Ellington, 1997) to examine their kinematics. They investigated that the average stroke of wing beat was around 90°. The average stroke amplitude can be predicted utilizing the stroke amplitude at midspan position $\frac{L}{2}\sin45{}^{\circ}$ as in flapping flight, where *L* is the wing length. In plunging movement, we selected the average stroke amplitude in flapping flight as our plunging amplitude. Consequently, $h=\;\frac{L_{fore}}{2}\sin45{}^{\circ}$ was set based on the length of forewing *L*_*fore*_. The pitch amplitude was set at 30° according to the kinematics measured on the actual dragonflies by Chen et al. (Chen et al., 2013).

**FIGURE 8:**
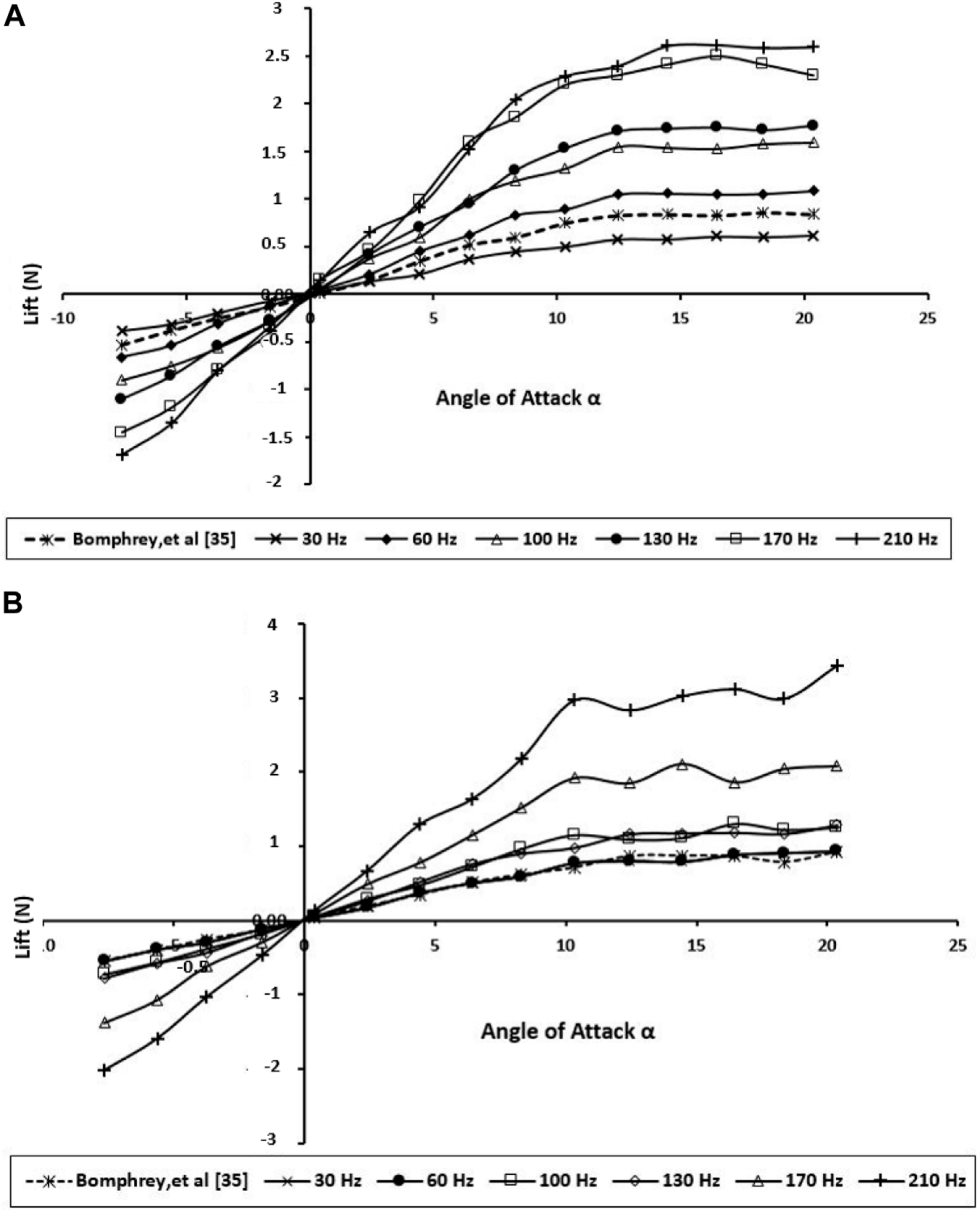
Generated lift for the BMAV’s wing at various angles of attack and wing beat frequencies; **(A)** forewing; **(B)** hindwing.

**FIGURE 9:**
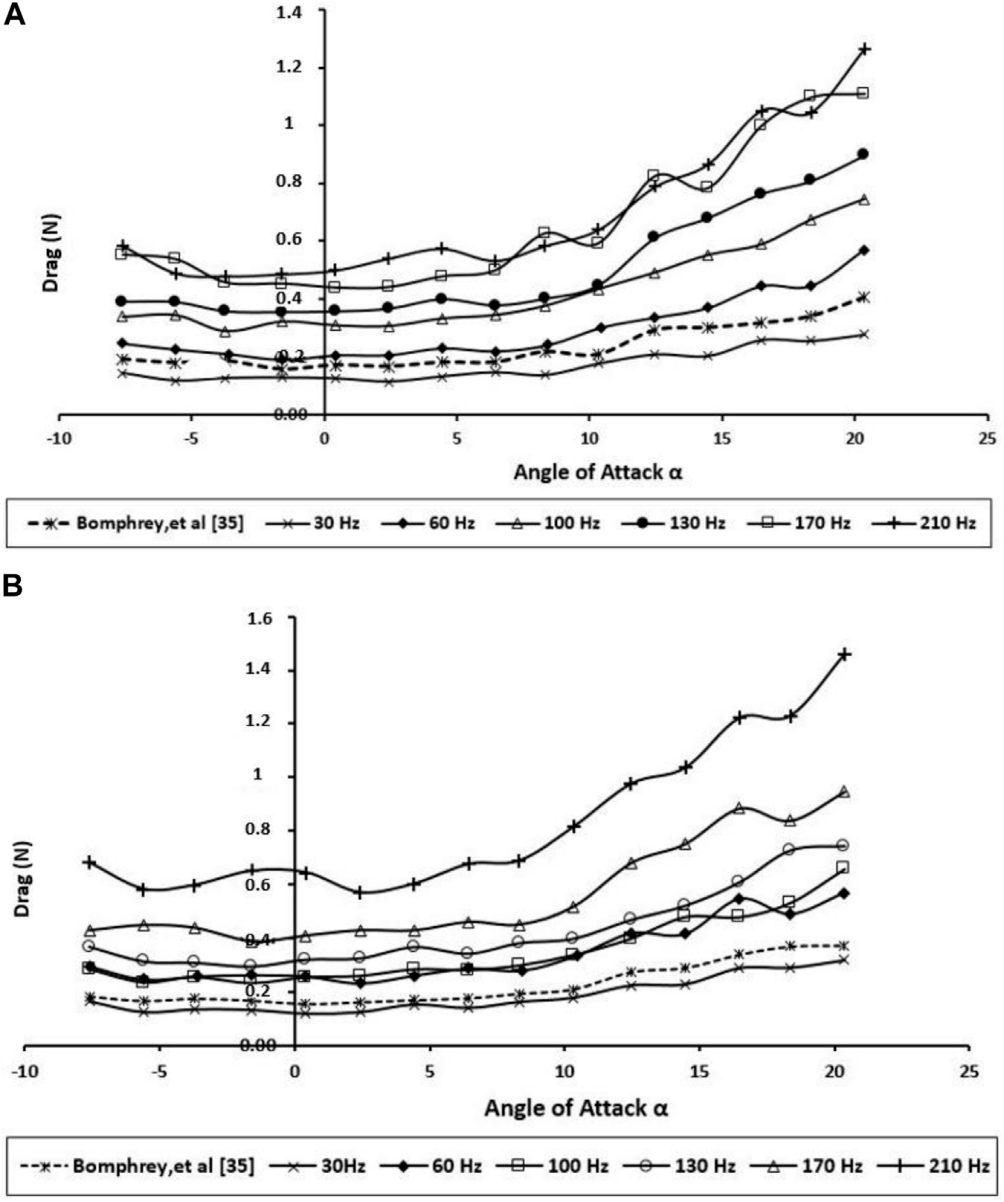
Produced drag for the BMAV’s wing at various angles of attack and wing beat frequencies; **(A)** forewing; **(B)** hindwing.

The aerodynamic performance of the dragonfly wings was examined experimentally by calculating the lift and drag forces for various angles of attacks and various wing beat frequencies as shown in Figures 8, 9, respectively. More pressure differences in the surface of the dragonfly wing had been generated by higher AOA. Consequently, the lift increases with the angle of attack. In addition, for the positive AOA, the drag increases with the angle of attack. As shown in Figures 8, 9, the current study’s results are in good agreement with those of Bomphrey and Nakata (Bomphrey et al., 2016).

According to Figure 8, the higher frequency creates a slightly greater lift force than the lower frequency at all AOAs up to 10, so the bibliometric corrugated wing performs better due to more gradual stall characteristics. This AOA might be higher than what dragonflies naturally use when gliding; nevertheless, this specification could enhance the stability during flapping flight.

The experiments demonstrate that when the wings are about midway through its downstroke, the lift was mostly influenced. This is acceptable because a higher lift was generated during this period, as the wings achieved the maximum flapping velocity around midway through the downstroke. Unsurprisingly, Figure 9 displays the hindwing lift was decreased by wing interaction at most wing beat frequencies. The reduction of the hindwing lift was not as remarkable as the one reported by Maybury and Lehmann (Maybury and Lehmann, 2004). This phenomenon can be explained as a downward flow is created in producing vertical force on the forewing, and the hindwing moves through the downwash-velocity field, which can cause a reduction in its aerodynamic forces.

The drag force (Figure 9) clearly shows that the drag performance of the BMAV wing at natural wing beat frequency is close to the real dragonfly wing even at a higher angle of attack (AOA ≥10.0°). Due to the flow separation on the lifting surface and stall, the drag force has increased dramatically. While the hindwing has its stall between 10.0° and 12.0° AOA, stall angles on flexible forewings were not found until between 15.0° and 17.0°. Compared with the various wing beat frequencies, the drag force acting on higher frequencies (170 and 210 Hz) was found to be the highest of all. By having flexible surface area, very high frequency causes the BMAV wing to have very poor resistance to adverse pressure gradient by flapping at higher AOA. This can also be found in the lift performance displayed in Figure 9 (b). The lift performance of the BMAV hindwing at high frequency is also found to be very unstable. Accordingly, they would not be very practical frequencies for BMAV due to the very poor stability.

**FIGURE 10:**
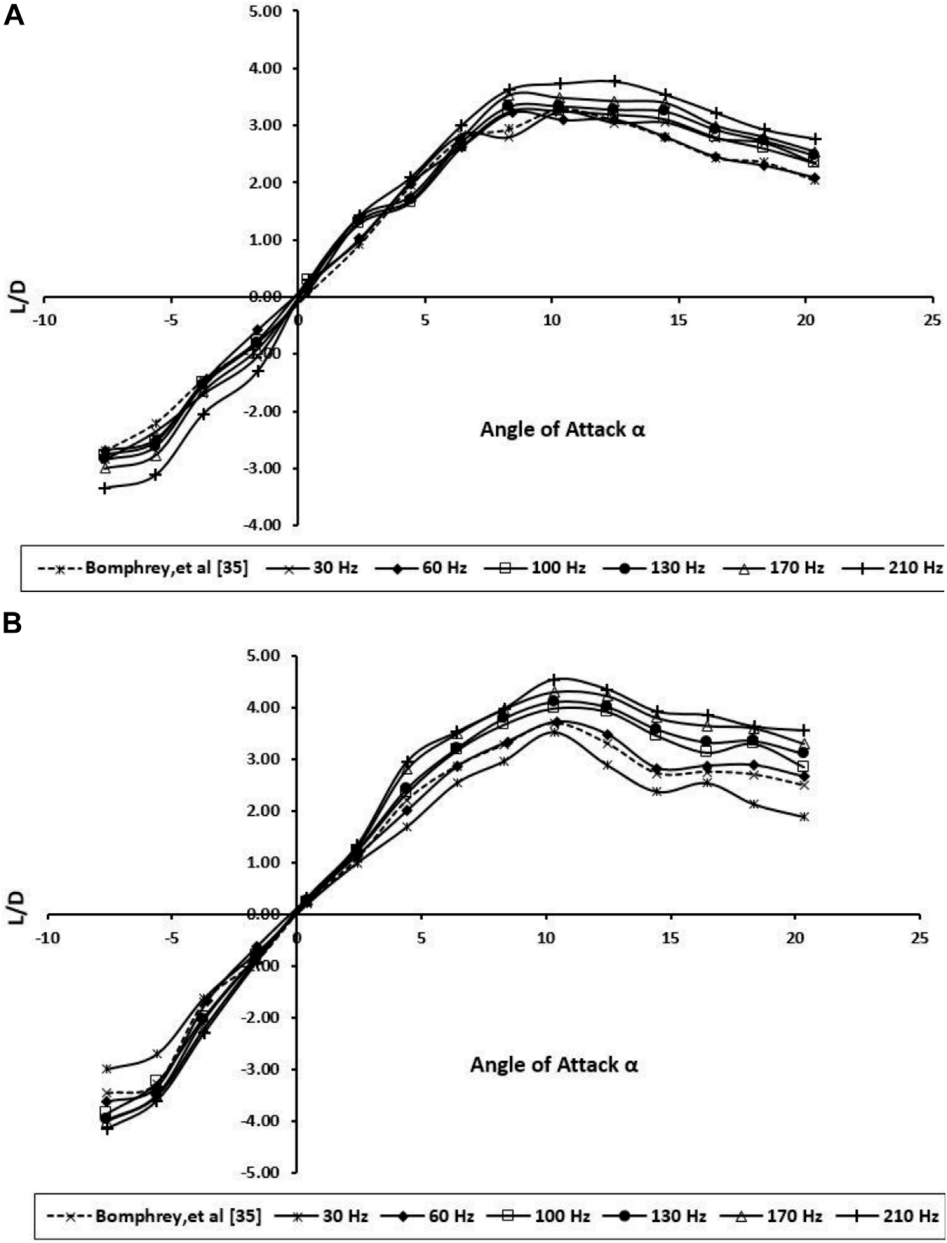
Lift-to-drag ratio for the BMAV’s wing at various angles of attack and wing beat frequencies; **(A)** forewing; **(B)** hindwing.

Drag, however, increases monotonically with increment in wing beat frequency (Figure 9). The result shows an enhancement in the lift-to-drag ratio with increasing wing beat frequency. Notably, based on Figure 10, the wing beat frequency does not have a significant effect on L/D improvement at low AOAs, which has been observed for high AOAs.

As mentioned in previous sections, actual scale BMAVs raises resistance to bending loads without significantly increasing compromising torsional stiffness or material volume, but as a conclusion, this is not offset by a substantial aerodynamic cost and may even lead to higher aerodynamic performance by enabling higher–aspect ratio geometries.

In dragonfly flight, the forewing and hindwings do not flap individually but interact with each other. The BMAV wings have been manually fitted in tandem configuration to run trials. The experiments were performed at various wing beat frequencies (30–210 Hz) with angles of attacks ranging from -10 to 20.

The forewing places in a positive pressure field created by the hindwing and consequently experiences reduced drag; on the other hand, the hindwing suffers greater drag due to the forewing. Combined aerodynamic performance is relatively good, especially in terms of the low drag owing to the wing high aspect ratios. Despite the fact that it is not acceptable to put the two BMAV wings excessively near one another (as the effective aspect ratio is reduced), a real dragonfly keeps the efficiency of each wing high by varying the wing angles to high efficiently (dashed line in Figure 10). In conventional, fixed-wing aircraft, high–aspect ratio wings reach better L/D at the cost of maneuverability.

**FIGURE 11:**
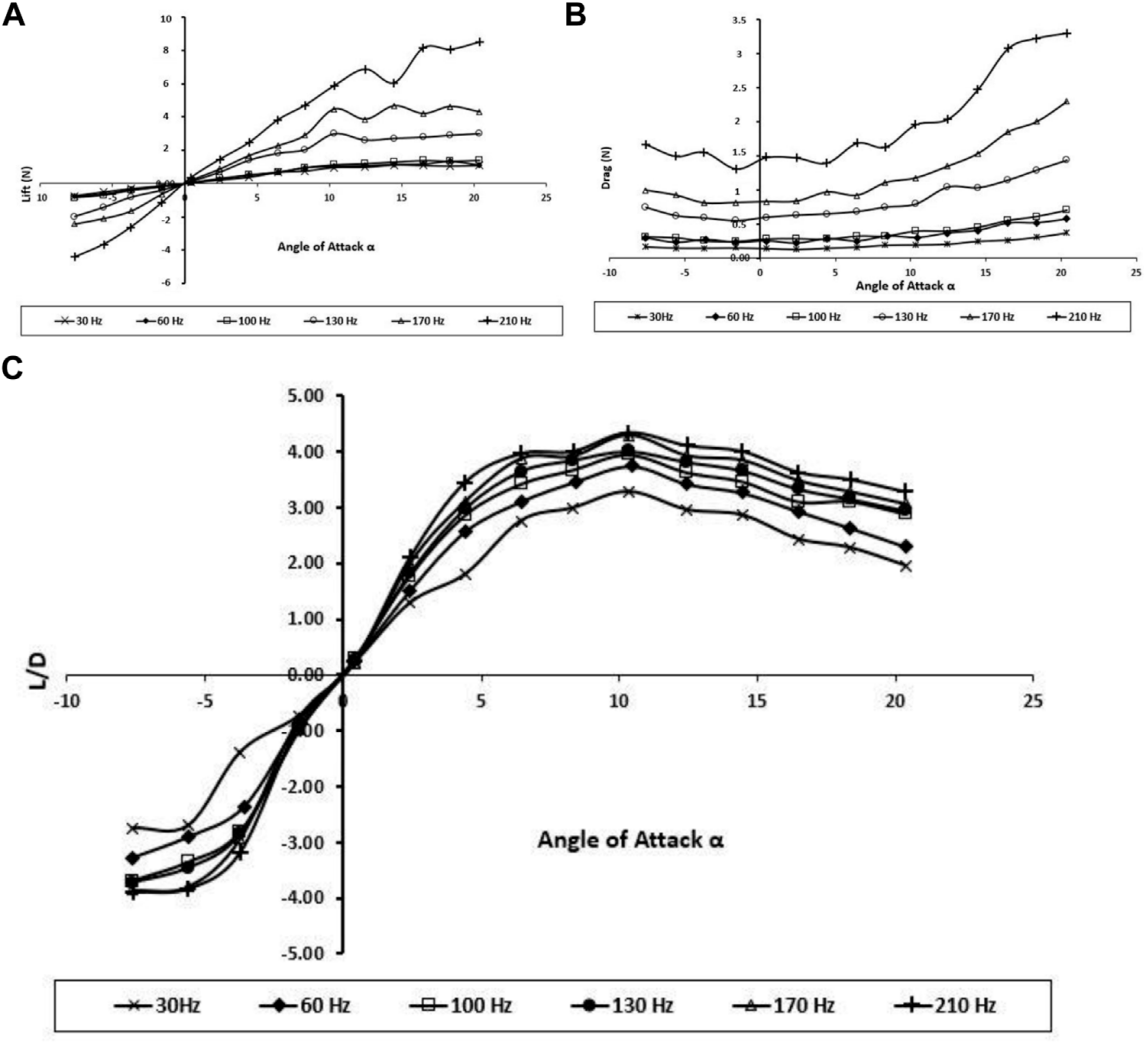
Effect of tandem wings on **(A)** Lift force; **(B)** drag force; and **(C)** lift-to-drag ratio.

Overall, the vortex interactions between the fore and hind wings can be considered either positive or negative. When the hindwing flaps with 0° of phase lag, constructive vortex interaction happens which strengthen the LEV generation of the hindwing, leading to large LEVs, and the resulting LEVs improve the peak lift and thrust production, as can be seen in Figure 11.

Furthermore, these experiments show that the tandem wings generate higher lift and drag forces than forewing and hindwing individually, and the aerodynamic performance of tandem wing results also shows improvement in the L/D and wing stability.

## Conclusion

The advantages of using BMAV wings are clearly presented in this article. The mechanism presented in this article generates approximately 50% higher lift and drag forces than the forewing and hindwing individually; the aerodynamic performance of tandem wing results also show improvement in the L/D and wing stability. The aerodynamic forces and aerodynamics performance were measured for the flapping wing configuration from −10° to 20° AOA at various wing beat frequency ranges from 30 to 210 Hz. The wing movement produces two distinct force peaks at the beginning and end of each flapping cycle that are consistent and illustrates the wake capture. In summary, direct measurements of the forces generated by flapping wings propose that the aerodynamics of dragonflies may be described by an interactive mechanism and the interaction of the fore and hindwing. The experiments demonstrate that the lift was mostly influenced when the wings are almost midway through its downstroke. In addition, they show that higher AOA produces higher pressure differences in the surface of the dragonfly wing. Consequently, the lift rises with the AOA. In addition, for the positive AOA, the drag increases with the angle of attack. In dragonfly flight, the forewings and hindwings do not flap separately but interact with one another. The forewing places in an area of positive pressure created by the hindwing and, therefore, experiences reduced drag; conversely, the hindwing suffers higher drag because of the forewing. Overall, the vortex interactions between the forewings and hindwings can be considered either positive or negative. When the hindwing flaps with 0° of phase lag, constructive vortex interaction happens, which strengthens the LEV generation of the hindwing, leading to large LEVs and the resulting improvement in the peak lift and thrust production.

## Data Availability

The original contributions presented in the study are included in the article, further inquiries can be directed to the corresponding authors.
